# Liver Abscesses as a Sign of Chronic Granulomatous Disease in Adolescent

**DOI:** 10.7759/cureus.17467

**Published:** 2021-08-26

**Authors:** Hussain A Al Ghadeer, Fadi N Busaleh, Jaber A Al Habeeb, Rabab M Alaithan, Abdullah E Almutahhar, Murtadha M Bin Abd, Mishael M Aldawood

**Affiliations:** 1 Pediatrics, Maternity and Children Hospital, AlAhsa, SAU; 2 Allergy and Immunology, Maternity and Children Hospital, AlAhsa, SAU

**Keywords:** chronic granulomatous disease, primary immunodeficiency disorder, liver abscess, x-linked genetic diseases, nadph oxidase deficiency

## Abstract

Chronic granulomatous disease (CGD) is an inherited primary immunodeficiency disease caused by a genetic defect in the nicotinamide adenine dinucleotide phosphate oxidase (NADPH) complex that affects phagocytes. This leads to recurrent severe bacterial and fungal infections manifested by recurrent pneumonia, also involving soft tissue, bones, and liver. Usually, CGD is presented and diagnosed in the first five years of life. In this case report, we describe a late presentation in an adolescent with multiple liver abscesses, the approach of diagnosis, and management.

## Introduction

Chronic granulomatous disease (CGD) is described as a rare primary immunodeficiency disease (PID) caused by a defect in one of the five subunits of the nicotinamide adenine dinucleotide phosphate oxidase (NADPH) complex, which impairs respiratory burst. Two-thirds of cases are inherited as X-linked [1]. The incidence of CGD is estimated to be one in every 200,000 to 250,000 live births [2]. The majority of cases are diagnosed early before five years of age, although the diagnosis can be made at any age. As a result of the genetic defect that impairs phagocyte function, patients with CGD are at high risk for recurrent life-threatening catalase-positive bacteria and fungal infections. The most common organisms are Staphylococcus aureus, Burkholderia onionensis, Serratia mucilage, Nocardia, and Aspergillus, which affect the skin, lymph nodes, bone, and liver [3]. This report describes a child with the later presentation of CGD through persistent fever and a liver abscess.

## Case presentation

This is a 12-year-old Saudi boy presented to Maternity and Children's Hospital - Alhassa in Saudi Arabia with a history of intermittent fever that initially responded to antipyretics but after few days the fever became continuous without response to antipyretics and antibiotics. It was associated with intermittent cough, non-productive, and without diurnal variation. Also, it was associated with weight loss around 8 kilograms, anorexia, epigastric pain non-specific, with episodes of vomiting. There was no history of recurrent infections, bone pain, hair loss, skin rash, or family history of immunodeficiency or haematological disorders like sickle cell anaemia. There was a history of the previous admission about a year ago with lobar pneumonia and parapneumonic effusion that responded well to 10 days course of antibiotic therapy. On examination, the patient looked ill, was lethargic without pain or dyspnea, febrile 38.6°C, HR: 100 beats/min, RR: 25 breaths/min, BP: 112/62 mmHg, O_2_ saturation: 98% on room air. Weight was 22 kg below the third percentile. There is decreased air entry in the right lower chest, the rest of the exam was unremarkable. Laboratory findings (Table 1) revealed leukocytosis (neutrophilia) with microcytic hypochromic anaemia and hypoalbuminemia with a normal hepatic renal profile.

**Table 1:** Laboratory and imaging investigations

Laboratory Test	Patient Result
Complete Blood Count	
Haemoglobin	8.5
	Reference Level: 11.5-13.5
White Blood Cells	14.92
	Reference Level: 4.5-13.5*10^3^
Platelets	415
	Reference Level: 150-350*10^3^
Blood Chemistry Tests	
Total Bilirubin (TBIL)	0.6
	Reference Level: 0.00 to 1.00 mg/dL
Direct Bilirubin (DBIL)	0.43
	Reference Level: 0.00 to 0.20 mg/dL
Albumin	19
	Reference Level: 34 to 54 g/L
Aspartate Aminotransferase (AST)	32
	Reference Level: 15 to 37 IU/L
Alanine Aminotransferase (ALT)	22.8
	Reference Level:0.00 to 35 IU/L
	Inflammatory Makers
Erythrocyte Sedimentation Rate (ESR)	101
	Reference Level: 0 and 20 mm/hr
Coagulation Profile	
Prothrombin Time (PT)	14.9
	Reference Level: 12.7 to 16.1 seconds
Partial Thromboplastin Time (PTT)	43
	Reference Level: 33.9 to 46.1 seconds
International Normalized Ratio (INR)	1.16
	Reference Level: 0.79 to 1.30
Imaging	
Chest X-ray	Right lower lung opacity
Abdominal Ultrasound	Multiple hepatic abscess
	-
CT Scan Abdomen and chest	linear atelectasis in both lungs with left lung solitary pulmonary nodule and multifocal hepatic pyogenic abscesses

**Figure 1:**
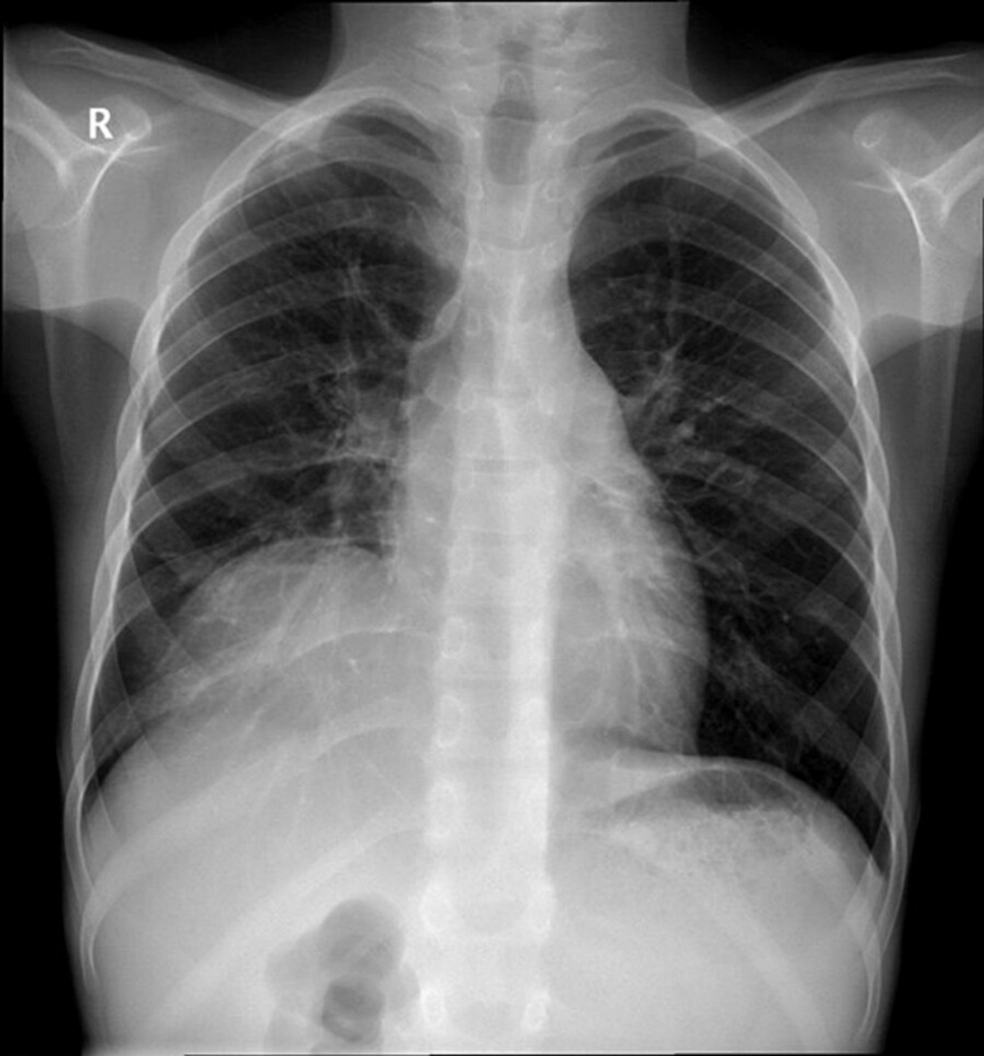
Chest x-ray, AP view, showing opacity in the right lower lung with vascular markings, normal cardiothoracic shadow with no obliteration of the costophrenic angles.

**Figure 2:**
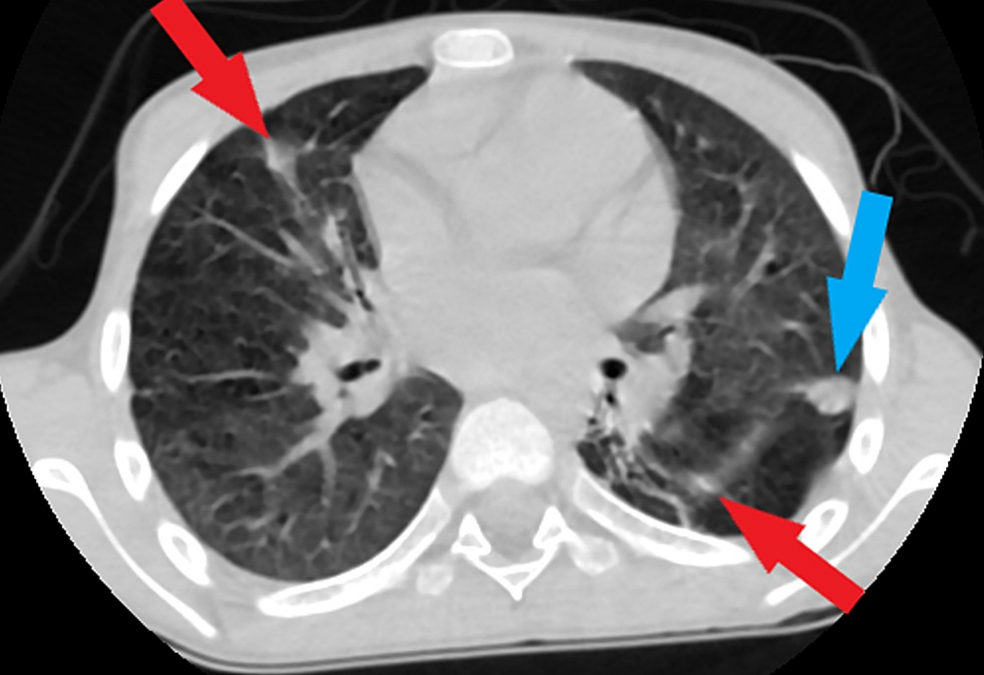
CT scan of the chest, axial view with no contrast showing multiple linear opacities in both lungs suggestive of bilateral linear atelectasis (red arrows) and a single solitary lung nodule in left lung measures 10*11 mm (blue arrow).

**Figure 3:**
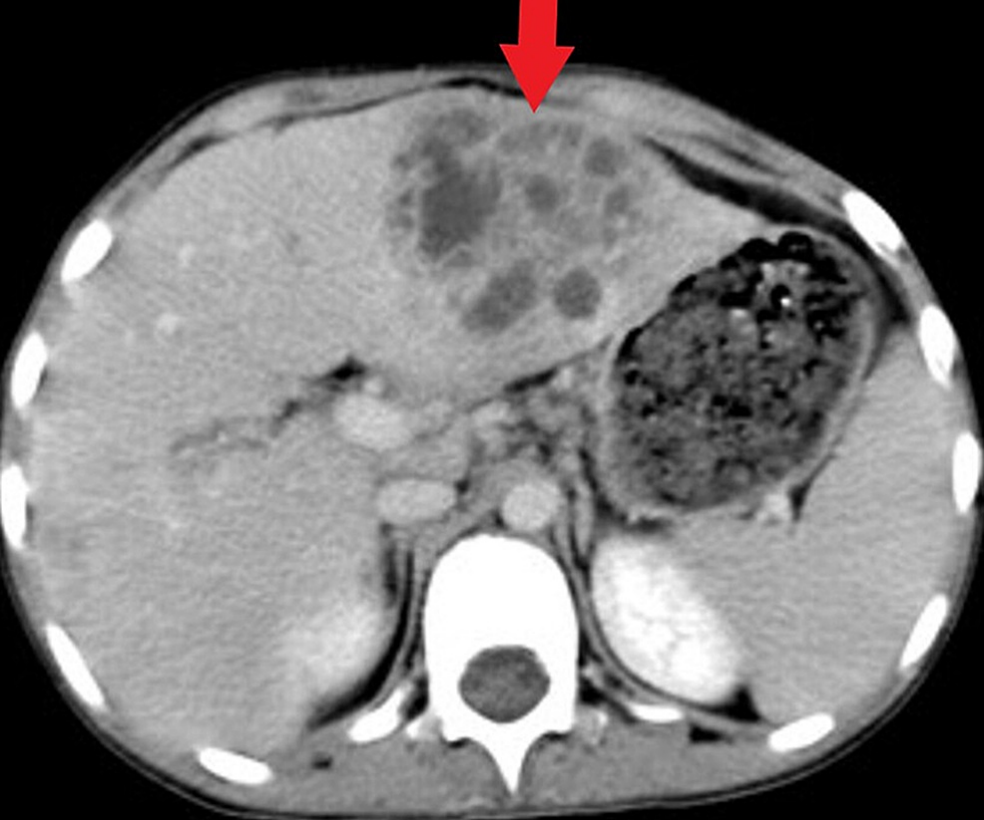
CT scan of the abdomen, axial plane with no contrast showing multiple cystic lesions in the left liver lobe (subcapsular in location) grape-like suggestive of hepatic abscesses.

Chest x-ray (Figure 1) initially showed infiltration of the right lower lobe. Abdominal Ultrasound showed multiple liver abscesses. Blood culture was obtained, and a broad-spectrum antibiotic was started covering gram-positive and negative organisms as well as anaerobes. A computed tomographic study (CT) of the chest and abdomen (Figures 2, 3) showed linear atelectasis in both lungs with a solitary nodule in the left lung and multifocal pyogenic liver abscesses. The liver abscess was drained by ultrasound-guided procedure and the drained pus was cultured and showed S. aureus. The patient was treated with culture-sensitive antibiotics for 10 days. Since pulmonary infection and unusual abscess site were noted, oxidative burst test and immunoglobulin level were requested to roll out immunodeficiency disorders. The result was positive for the oxidative burst test and a normal immunoglobulin level. Based on the clinical features and investigations, a diagnosis of CGD was made. The patient was discharged home with prophylactic antibiotics and antifungals and seen regularly by an immunologist.

 

 

## Discussion

The NADPH oxidase complex consists of five proteins: gp91phox, p22phox, p47phox, p67phox, p40phox, and a sixth protein EROS as a stabilizer [4]. Any defect in any of these proteins can lead to CGD. Approximately 65%-70% of cases are inherited as X-linked due to mutations in the CYBB gene, which encodes gp91phox. CGD can also be inherited in an autosomal recessive manner when the mutations occur in the NCF1, NCF2, CYBA, and NCF4 genes, which encode p47phox, p67phox, p22phox, and p40phox, respectively [5]. In the last decade, the survival rate has improved significantly, allowing more than 50% of patients with CGD to live at least until the age of 25 years, whereas 60 years ago the survival rate was less than 40% [6,7]. Although the survival rate has improved, it depends on the clinical patterns, mode of inheritance, as X-linked inheritance is more severe and the mortality rate is higher, which defect occurs at which gene, and the type and location of the mutation. All these factors may play an important role in the survival rate [8,9].

CGD is characterized by recurrent life-threatening bacterial and fungal infections that occur due to the impairment of the enzyme NADPH oxidase. The majority of cases are caused by catalase-positive organisms. The most common organisms are S. aureus, Serratia, Burkholderia, Pseudomonas as bacteria and Aspergillus and Candida as fungi [10]. In this case, the culture was positive for S. aureus. Also, as a result of prolonged inflammatory response due to defect in oxidant production by phagocytes leading to granuloma formation [11]. The pattern of presentation varies from patient to patient. Globally, recurrent pulmonary infections are the main symptoms followed by soft tissue infections “lymphadenitis”, which is most common in Middle Eastern countries. Our patient had persistent fever and liver abscess, which in most cases occurred as later complications and were not the usual presenting sign. Primary Immunodeficiency disorders should be considered when there are red flags warning of immunodeficiency investigations (Table 2) [12]. CGD is considered when there is recurrent pulmonary infection or abscesses in unusual locations [13].

Although CGD is rare, it should be diagnosed if strongly suspected. Nitroblue tetrazolium test (NBT) is usually reduced to formazan. In CGD, NBT is not reduced due to a failure of superoxide formation. Dihydrorhodamine-1,2,3 assay (DHR) is a flow cytometric analysis that evaluates NADPH activity. DHR is more sensitive [14,15].

Early diagnosis of CGD is critical for effective treatment and good outcomes. Acute infection should be treated aggressively with antibiotics, followed by prophylactic antibiosis with sulfamethoxazole-trimethoprim and antifungal azoles, which significantly reduces morbidity and mortality. Curative therapy for CGD is by hematopoietic stem cell (HSC) transplantation. For those who are not human leukocyte antigen (HLA) identical, genetic modification of autologous HSCs can be used as an alternative therapy [16].

**Table 2:** Red flags for primary immunodeficiency disorders

Red flags for primary immunodeficiency disorders
Recurrent infection
Invasive infection
Atypical pathogens
Partial response to antibiotic treatment or prolonged course
Failure to thrive
Chronic diarrhea
Fungal infections, unexplained and unusual in site
Unexplained skin rash
Family history of immunodeficiency

## Conclusions

Infections are essential for the development of children’s immune systems. They serve as a battleground for the formation and recruitment of children's immune systems. These infections vary with their degree, but they have a range of acceptability and the consequences when they go beyond suggest immune deficiency disorders. Recurrent lung and soft tissue abscesses are the main signs of CGD, but not the only ones. The presence of an abscess, in which S. aureus is growing, is highly suggestive of an NADPH oxidase complex defect called CGD. Usually seen in the first decade of life especially the first half but the delayed presentation is reported. The management of this is aimed at treatment of current infection, but usually, the management of this patient is difficult, burden with complications that required surgical intervention and prophylaxis of subsequent infection. Outcomes today are favourable due to the development of hematopoietic stem cell transplantation.
